# Evaluating Genioplasty Procedures: A Systematic Review and Roadmap for Future Investigations

**DOI:** 10.3390/cmtr18010005

**Published:** 2025-01-03

**Authors:** Sebastiaan W. R. Dalmeijer, Tom C. T. van Riet, Jean-Pierre T. F. Ho, Eddy (A. G.) Becking

**Affiliations:** Amsterdam University Medical Center, Department of Oral and Maxillofacial Surgery, Meibergdreef 9, 1105 AZ Amsterdam, The Netherlands; t.c.vanriet@amsterdamumc.nl (T.C.T.v.R.); j.p.ho@amsterdamumc.nl (J.-P.T.F.H.);

**Keywords:** genioplasty, orthognathic surgery, patient outcome assessment, treatment outcome

## Abstract

Study design: Systematic review. Objective: This systematic review examines the existing literature concerning the objective and subjective evaluations of osseous genioplasty outcomes. Methods: A comprehensive search was conducted in databases including PubMed, Embase, and Web of Science, yielding 2563 references, which were screened by two independent reviewers. We included 105 articles originating from 25 different countries. Data were systematically extracted, categorized, and documented. Results: Genioplasty was performed in 5218 patients, either independently (3560 cases) or in combination with other orthognathic procedures (1696 cases), with a predominant focus on female patients (64%). Objective evaluation primarily focused on surgical accuracy, relapse, and neurosensory disturbance, while subjective assessments were largely related to aesthetics and patient satisfaction. Despite significant advancements in three-dimensional surgical planning and assessment, the review highlights a lack of standardized methods for evaluating isolated genioplasty outcomes. Conclusions: The findings emphasize the need for improved and validated instruments that specifically assess the functional and aesthetic results of genioplastic surgery. Future research should prioritize patient-centered prospective studies and the development of assessment tools to ensure more comprehensive and reliable outcome evaluations.

## 1. Introduction

### 1.1. The Chin and Genioplasty

The chin, or mental protuberance, constitutes the most visible and prominent aspect of the lower facial third, playing a crucial role in facial balance and overall harmony [1,2,3]. It significantly contributes to an individual’s facial identity [4]. The perception of the chin’s aesthetics varies depending on factors such as gender, ethnicity, cultural background, standards, and prevailing fashion trends [5,6]. Dentofacial deformities encompass a range of conditions involving structural abnormalities in the alignment and positioning of teeth, jaws, and facial bones, which may necessitate orthodontic, oral, and maxillofacial surgical treatments to address functional and aesthetic concerns. They can substantially alter facial attractiveness and impact individuals’ psychological perception of their facial appearance. Notably, approximately 75 percent of dentofacial deformities manifest in the lower facial third [7]. Orthognathic surgery represents a valuable avenue for enhancing facial aesthetics and addressing imbalances. Within this context, both alloplastic and osseous genioplasty procedures are used to, surgically, correct chin deformities. Genioplasty can be performed either independently to camouflage a dentofacial deformity or in conjunction with other orthognathic procedures. The indications for genioplasty encompass functional issues (e.g., obstructive sleep apnea or inadequate lip closure), dentofacial deformities, and aesthetic reasons (e.g., profiloplasty or feminization) [8].

### 1.2. Planning and Evaluation

Orthognathic surgery planning has evolved significantly since its inception, shifting from traditional two-dimensional (2D) X-rays and cephalometric analysis [9] to more contemporary three-dimensional (3D) virtual surgical planning, offering improved predictability and precision [10,11,12,13,14]. This transition to 3D planning has brought both objective and subjective evaluation to the forefront. Objective evaluation now often encompasses surgical accuracy assessments in three dimensions using tools like the orthognathic analyzer. Subjective evaluations have seen the proliferation of various questionnaires and tests [15]. In contrast, while genioplasty has also embraced three-dimensional planning, the literature reveals a striking absence of clear guidelines for evaluating the outcomes of genioplasty. Genioplasty often remains overshadowed by the effects of other concurrent orthognathic osteotomies [12]. The subjective evaluation of genioplasty outcomes predominantly utilizes questionnaires, such as Face-Q, Orthognathic QoL, the pinprick test, Visual Analog Scale (VAS), Numerical Rating Scale (NRS), and various other survey tools. Nevertheless, these assessment methods often inadequately capture the chin’s distinct role in overall facial enhancement and can present challenges in isolating its individual impact [16,17,18,19].

As genioplastic surgery is often overshadowed by more extensive procedures in the scientific literature, a knowledge gap exists regarding the isolated outcomes of genioplastic surgery.

### 1.3. The Aim of This Study

This systematic review is designed to offer a comprehensive overview of objective and subjective evaluations of genioplastic surgery. The aim of this review is to explore the existing knowledge gap and to deliver guidelines for future research.

## 2. Materials and Methods

### 2.1. Methods

This review followed the guidelines established by both the Preferred Reporting Items for Systematic Reviews (PRISMA) and the Joanna Briggs Institute (JBI) [20,21]. The study protocol was registered on INPLASY (2024120117).The bibliographic databases PubMed, Embase, APA PsycINFO (Ebsco), and Web of Science (Core Collection) were searched on 9 August 2023. Articles in both English and Dutch languages were included. The search and removal of duplicate articles were carried out by a medical information specialist using Endnote X20.0.1 (Clarivate, Philadelphia, PA, USA), following the Amsterdam Efficient Deduplication (AED) method [22] and the Bramer method [23].

The search terms used included Genioplasty; Mentoplasty; Chinplasty; Micrognathism; Macrognathism; Prognathism; Retrognathism; and Orthognathic Surgery. The complete search strategies for all databases can be found in Appendix A.

### 2.2. Selection Process

Articles identified through the search strategy were imported into a web application for systematic reviews (Rayyan, Qatar Computing Research Institute, Doha, Qatar) [24]. Subsequently, two reviewers independently assessed all potentially relevant titles and abstracts for eligibility. Any discrepancies in judgment were resolved through a consensus procedure with the assistance of a third-party referee.

Studies were excluded during full-text screening if they did not allow for the separation of results related to osseous genioplasty only. For objective exclusion criteria, instances where a study described BSSO with genioplasty and only evaluated Pogonion were excluded. However, studies that individually analyzed osseous genioplasty, for example using the OrthoGnathicAnalyser 2.0 (3D MedX, 3D Lab Radboudumc, Nijmegen, The Netherlands) were included [12,13]. In terms of subjective criteria, studies using general questionnaires were excluded, while those specifically focusing on genioplasty were included. The primary outcome needed to be the evaluation of osseous genioplasty, not alloplastic genioplasty; otherwise, studies were excluded. Additionally, articles that did not present primary data and those involving animal or laboratory studies were excluded.

### 2.3. Data Assessment

The full texts of the selected articles were obtained for further examination. Data were extracted using a standardized data extraction form. Data concerning independent results of genioplasty, both subjective and objective, were collected. For averages, for example, if one study had 2 cases with a 10 mm advancement, and another study had 2000 cases with a 2 mm advancement, the unweighted average would be 6 mm. Additionally, data regarding basic patient and literature characteristics (e.g., age and study methodology), as well as basic surgical characteristics (e.g., fixation technique, either 2D or 3D), were collected.

## 3. Results

**Figure 1:**
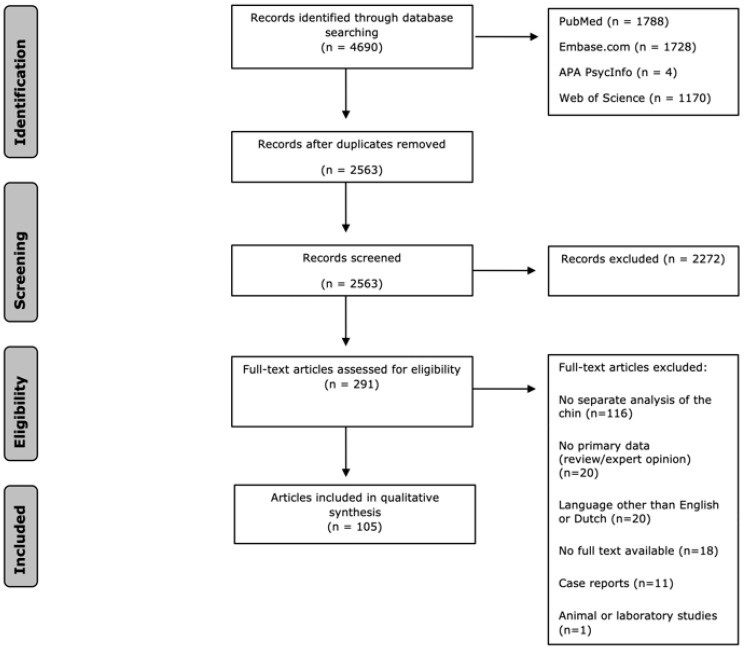
Prisma table of the search process and results.

The literature search yielded 4690 references. After removing duplicates, 2563 articles underwent screening based on their titles and abstracts. Following the title and abstract screening, 2272 articles were excluded. The remaining 291 articles underwent full-text assessment for eligibility, resulting in the inclusion of 105 articles (refer to Figure 1). The main exclusion criterion was the absence of a separate analysis of the chin.

### 3.1. Literature and Patient Characteristics

The countries of origin most frequently mentioned were the United States of America with 22 articles (21%), China with 12 articles (11%), India with 11 articles (10%), South Korea with 11 articles (10%), and France with 8 articles (8%). A minority of the studies had a prospective study design, accounting for 19 articles (18%).

The review encompassed 7691 cases, with the majority being females (4973). The reported mean age was 25 years old (ranging from 10 to 72 years). Out of the 5218 genioplasties performed, 3560 (68%) were performed as solitary procedures, while 1696 (33%) were combined with other orthognathic surgeries. The reported average follow-up period was 30 months, with a range spanning from 1 week [25,26] to 204 months [27,28].

### 3.2. Surgical Characteristics

**Figure 2:**
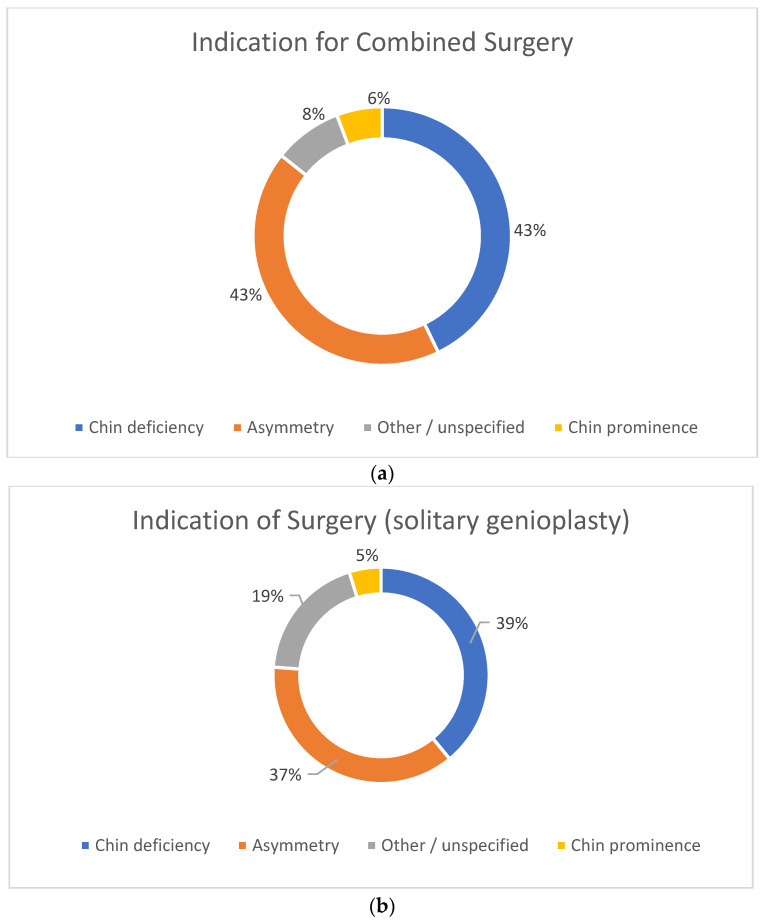
(**a**) Indication for surgery in both solitary and combined osseous genioplasty; chin deficiency, asymmetry, other/unspecified, chin prominence. (**b**) Indication for surgery in solitary osseous genioplasty; chin deficiency, asymmetry, other/unspecified, chin prominence.

Different indications for surgery were found and categorized in three main groups: chin deficiency, chin prominence, or asymmetry. Chin deficiency and facial asymmetry were found most frequently, both when combined with other procedures as in isolated genioplasties (Figure 2a,b). In both groups, chin prominence was a less common indication, seen in 6% of cases in the combined surgery group and 5% in the solitary genioplasty group.

#### 3.2.1. Surgical Movements

The reported chin movements, whether planned or realized, included advancement and setback, as documented in 44 articles (42%). In the combined group, the average chin advancement was 6.40 mm (range 0.96–18.0 mm), and the average setback was 3.13 mm (range 3.0–3.3 mm) [29]. In the solitary genioplasty group, the average advancement was 6.54 mm (range 4.18–8.39 mm). The average setback was 3.15 mm (range 3.00–3.30 mm).

#### 3.2.2. Fixation Techniques

The prevalent method of fixation in use is osteosynthesis plates, as reported in 61 articles (58%), with initial documentation dating back to around 1990 [30]. In 23 articles (22%), a combination of techniques was reported, while the specific fixation method was not specified in 13 articles (12%). The triple wire technique was reported five times (5%) [31,32,33,34,35], and lag screws were used in two cases (2%) [36,37].

The selection of fixation techniques changed over time, as illustrated in Figure 2. The triple wire technique was frequently observed in older articles published before 2000, whereas the use of osteosynthesis plates, often in combination with other techniques, became more prevalent after 2000.

### 3.3. Evaluation

In this review, both objective and subjective measurement methods were assessed. Objective analysis was conducted in 55 studies (52%), subjective analysis in 13 studies (12%), and a combination of both objective and subjective analysis in 37 studies (35%). Among the analyzed articles, 75 (71%) centered on the assessment of the chin in isolation, while 30 (29%) encompassed evaluations of the chin alongside other concurrent orthognathic procedures.

#### 3.3.1. Objective Evaluation

##### Objective Outcome Measures

**Figure 3:**
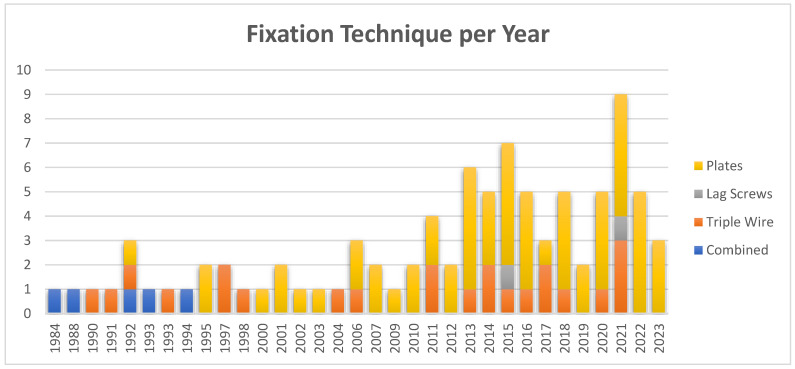
Fixation techniques per year (plates, lag screws, triple wire, and combined techniques: lag screws and triple wire).

In total, 92 articles (88%) included objective outcome parameters (see Figure 3). Among these, accuracy was the most frequently reported parameter, mentioned in 74 articles (70%). Relapse was reported in 17 articles (16%), followed by post-operative complications in 13 articles (12%), and surgery time in 10 articles (10%) [10,30,38,39,40,41,42,43,44,45]. The effect of genioplasty on the airway was discussed in eight articles (8%) [27,41,46,47,48,49,50,51], while neurosensory disturbance was addressed in four articles (4%) [43,52,53,54].

##### Surgical Accuracy

**Figure 4:**
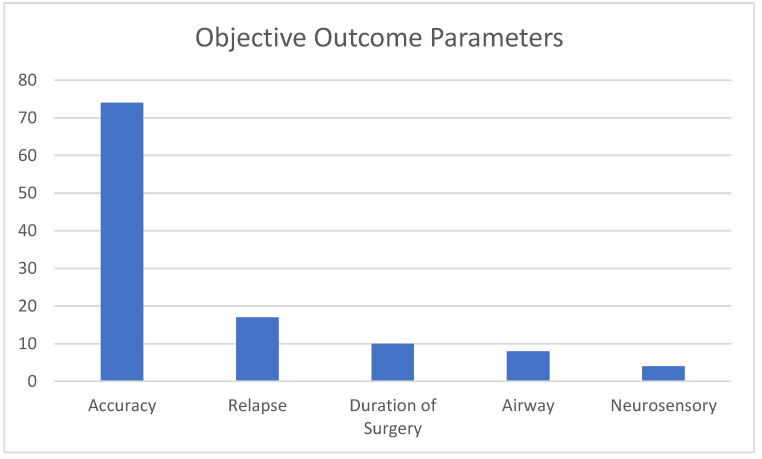
Objective outcome parameters.

The evaluation of the chin can be performed using 2D and 3D cephalometric analyses, or a combination of both. The majority of reviews emphasize surgical accuracy, with 74 articles (70%) focusing on this aspect. Specifically, 2D cephalometric analysis was mentioned in 65 articles (62%), while 3D analysis was discussed in 11 articles (10%). A combination of both methods was reported in 15 articles (14%). Notably, the evolution of evaluation methods is evident, as older articles displayed a higher propensity for utilizing 2D cephalometric analysis compared to publications from the past decade. An increase in the utilization of 3D computer analysis has been observed from 2010 onwards (see Figure 4).

From a radiographic perspective, surgical outcomes can be assessed at either the bony level, soft tissue level, or both simultaneously. A combination of bony and soft tissue level evaluation was used in 36 articles (34%). In 27 articles (26%), bone-level cephalometric points were analyzed, while 42 articles (40%) did not specify which points were used.

#### 3.3.2. Subjective Evaluation

Of the articles that conducted subjective evaluations, either independently or in conjunction with objective assessments, 27 articles (26%) employed questionnaires. Of these questionnaires, 24 (89%) had been validated for orthognathic surgery as a whole, while the rest were in-house designs (Table 1).

##### Aesthetic and Other Subjective Outcomes

**Figure 5:**
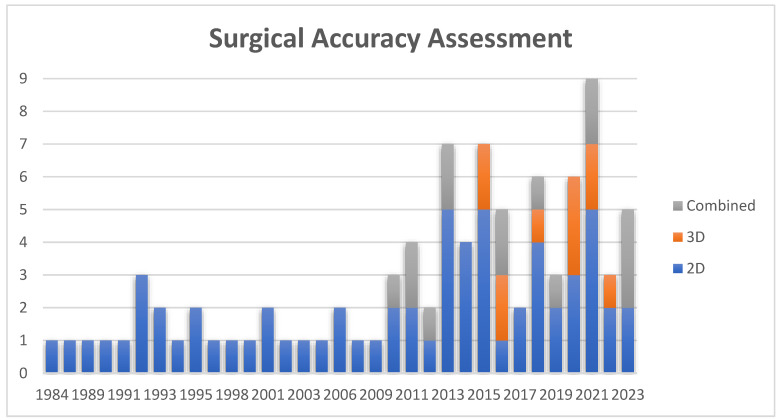
Methods for surgical accuracy assessment over the years.

The most frequently mentioned subjective outcomes were aesthetics and overall patient satisfaction, which were mentioned 40 times each (32%). In addition to aesthetic outcomes and patient satisfaction, the articles also addressed function, neurological disturbance, psychological well-being, postoperative pain, and surgical practicality (see Figure 5).

### 3.4. Complications

**Figure 6:**
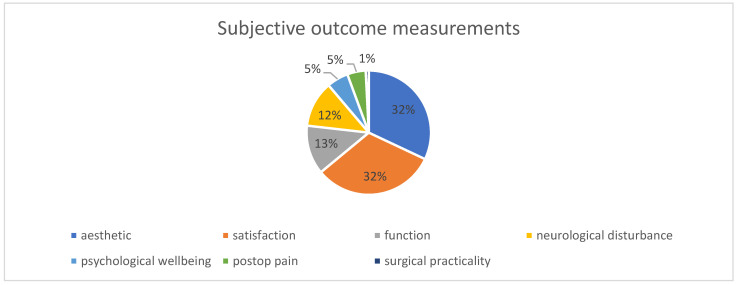
Subjective outcome measurements.

**Figure 7:**
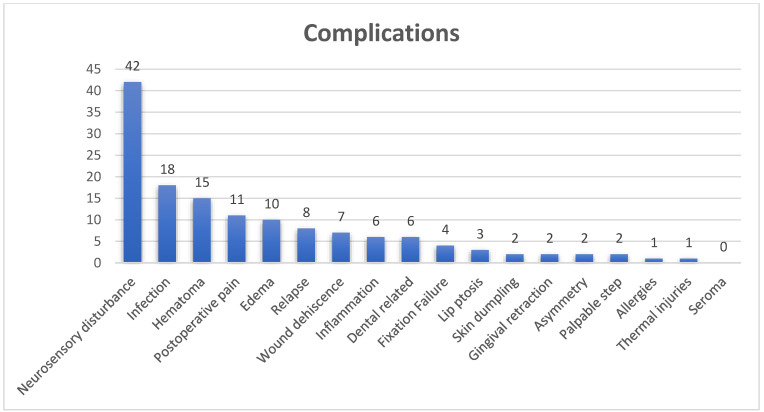
Complications mentioned.

In 57 articles (54%), multiple complications were discussed, while 48 articles (46%) made no mention of any complications. Among the complications noted, neurosensory disturbance was referenced in 42 studies (40%), followed by infection in 18 articles (17%), hematoma in 15 articles (14%), and post-operative pain in 11 articles (10%) (see Figure 6 and Figure 7).

## 4. Discussion

The primary objective of this systematic review was to examine the literature regarding the assessment of the chin following osseous genioplasty. Genioplasty was conducted in 5218 patients, either as a stand-alone procedure (3560 cases) or in conjunction with other orthognathic surgeries (1696 cases).

Surgical outcomes can be analyzed from both 2D and 3D perspectives, involving changes in both soft and bony tissues and their respective ratios. Traditional orthognathic planning predominantly relied on cephalometric analysis on plain 2D X-rays. However, the availability of Cone Beam Computed Tomography (CBCT) has led to a shift toward 3D virtual surgical planning, offering enhanced predictability and accuracy. Among the frequently examined outcome parameters, accuracy was addressed in 74 articles (70%).

Out of the 105 articles reviewed, only 27 evaluated subjective (patient-related) outcomes of genioplasties. It is common that questionnaires employed to evaluate subjective outcomes do not specifically target the chin, which may create difficulties in differentiating chin-related outcomes from the wider impact of facial corrections, especially simultaneous mandibular osteotomies. Subjective evaluation predominantly focuses on aesthetic outcomes, with limited attention given to other aspects like Quality of Life and psychological well-being, which are often underrepresented. The majority of questionnaires have been validated for general orthognathic surgery in general and lacks validation specifically for the chin surgery in isolation. Aesthetic outcomes and patient satisfaction are considered distinct results in our study, as authors use these terms separately. Patients may perceive the surgical result as visually pleasing or feel satisfied with functional improvements, such as enhanced lip closure. It is possible to find a result aesthetically appealing while experiencing sensory numbness, or to be satisfied with the functional outcome despite not finding it visually attractive. However, there is often considerable overlap between these two aspects.

Consequently, there exists an opportunity for further enhancement and advancement of questionnaires, such as the Face-Q Chin and Orthognathic Quality of Life (OQOL), to improve the subjective assessment of chin-related outcomes. This, in turn, can lead to a more detailed and comprehensive understanding of the results achieved through genioplasty procedures.

This study comes with certain limitations, primarily due to the prevalence of retrospective research and the lack of standardized evaluation methods, especially in the context of isolated genioplasty analysis. While it would have been preferable to include exclusively genioplasty-focused studies, doing so would have significantly limited the available data. A substantial heterogeneity in the inclusion criteria and surgical techniques across the studies was encountered, which may have influenced the outcomes. Unfortunately, an analysis of factors such as the differences between plates and screws could not be conducted due to the high number of included studies.

## 5. Conclusions and Recommendations for Future Research

In conclusion, the existing research landscape is characterized by a reliance on retrospective studies and a strong focus on objective surgical measures, particularly surgical accuracy. Subjective evaluation of genioplasty outcomes, especially from the patient’s perspective, has received comparatively less attention. This might be attributed to the frequent combination of genioplasty with other orthognathic surgeries, which can present challenges in conducting high-quality research studies.

Recommendations for future research in this field include a shift towards prospective studies with a heightened focus on the patient’s experience. The use of standardized, validated questionnaires with dedicated chin sections, focusing on both function and aesthetics, such as the Face-Q Chin or OQOL, can help improve the subjective evaluation process. Additionally, objective parameters for assessing the chin should be employed while minimizing the impact of other osteotomies, as demonstrated by tools like the Orthognathic Analyzer 2.0. Research can also benefit from established protocols. These efforts will contribute to a more comprehensive and reliable understanding of genioplasty outcomes, with particular attention to the chin’s role in facial harmony and overall patient satisfaction.

## Figures and Tables

**Table 1:** Subjective Outcome Questionnaires.

Authors	Title	Year	Description	Chin Mentioned	Validated
Bertossi, D.; Galzignato, P. F.; Albanese, M.; Botti, C.; Botti, G.; Nocini, P. F.	Chin Microgenia: A Clinical Comparative Study	2015	Global Aesthetic Improvement Scale (GAIS)	no	yes
Chan, D.; Ducic, Y.	A Simplified, Reliable Approach for Advancement Genioplasty	2016	Patient Satisfaction Survey	no	yes
Chang, E. W.; Lam, S. M.; Karen, M.; Donlevy, J. L.	Sliding genioplasty for correction of chin abnormalities	2001	Patient Satisfaction Survey	no	yes
Choi, B. K.; Yun, I. S.; Kim, Y. S.; Roh, T. S.; Park, S. E.; Bae, J. Y.; Jung, B. K.	Effects of Hat-Shaped Mortised Genioplasty with Genioglossus Muscle Advancement on Retrogenia and Snoring: Assessment of Esthetic, Functional, and Psychosocial Results	2019	Esthetic and Surgical Outcome	yes	yes
Deshpande, S. N.; Munoli, A. V.	Osseous genioplasty: A case series	2011	Patient Satisfaction Survey	yes	yes
Frapier, L.; Picot, M. C.; Gonzales, J.; Massif, L.; Breton, I.; Dauvilliers, Y.; Goudot, P.;	Ventilatory disorders and facial growth: benefits of early genioplasty	2011	Patient Satisfaction Index	yes	yes
Fu, X.; Qiao, J.; Rui, L.; Liu, J. F.; Gui, L.	A Novel Method to Attain Highly Symmetric Oblique Mandibular Chin-Body Osteotomy	2015	Patient Satisfaction Survey	yes	yes
Guyuron, B.; Raszewski, R. L.	A critical comparison of osteoplastic and alloplastic augmentation genioplasty	1990	Questionnaire	yes	yes
Jacks, S. C.; Zuniga, J. R.; Turvey, T. A.; Schalit, C.	A retrospective analysis of lingual nerve sensory changes after mandibular bilateral sagittal split osteotomy	1998	Nerve Sensation Questionnaire	no	yes
Jiang, Y.; Yang, B.; Yang, F.; Li, B.; Ma, H.; Huang, Q.; Sun, T.; Lei, B.; Shuang, K.	One-Half Wedge Osteotomy Genioplasty for Correction of Chin Deviation Based on Three-Dimensional Computed Tomography Measurements and Simulation	2021	Postoperative Satisfaction Questionnaire	yes	no
Khalifa, G. A.; Mohamed, F. I.	Aesthetic outcomes and morphological changes in chin parameters after mandibular distraction and subsequent advancement genioplasty in patients with mandibular micrognathia	2018	Postoperative Satisfaction Questionnaire	yes	yes
Olkun, H. K.; Borzabadi-Farahani, A.; Uckan, S.	Orthognathic Surgery Treatment Need in a Turkish Adult Population: A Retrospective Study	2019	Index of Orthognatic Functional Need (IOFTN)	yes	yes
Oth, O.; Mestrallet, P.; Glineur, R.	Clinical Study on the Minimally Invasive-Guided Genioplasty Using Piezosurgery and 3D Printed Surgical Guide	2020	Surgical Guide Satisfaction Questionnaire	no	no
Raffaini, M.; Magri, A. S.; Agostini, T.	Full Facial Feminization Surgery: Patient Satisfaction Assessment Based on 180 Procedures Involving 33 Consecutive Patients	2016	Facial Feminization Surgery Outcomes Evaluation Questionnaire	no	no
Roul-Yvonnet, F.; Quilichini, J.; Leyder, P.	Split-Overlapping Genioplasty: Surgical Technique, Morphological and Radiological Long-Term Outcomes	2017	Surgeon and population Satisfaction	yes	no
Schwitzer, J. A.; Albino, F. P.; Mathis, R. K.; Scott, A. M.; Gamble, L.; Baker, S. B.	Assessing Patient-Reported Outcomes Following Orthognathic Surgery and Osseous Genioplasty	2015	Face-Q	yes	yes
Singh, S.; Mehrotra, D.; Mohammad, S.	Profile changes after conventional and chin shield genioplasty	2014	Patient Satisfaction Score	yes	no
Tawa, P.; Brault, N.; Luca-Pozner, V.; Ganry, L.; Chebbi, G.; Atlan, M.; Qassemyar, Q.	Three-Dimensional Custom-Made Surgical Guides in Facial Feminization Surgery: Prospective Study on Safety and Accuracy	2021	Patient Satisfaction Questionnaire	yes	yes
Troedhan, A.	Piezotome Genioplasty Reduces Postsurgical Morbidity and Enhances Patient Satisfaction: A Randomized Clinical Trial	2018	Genioplasty Outcome Evaluation (GOE)	yes	yes
Troulis, M. J.; Kearns, G. J.; Perrott, D. H.; Kaban, L. B.	Extended genioplasty: long-term cephalometric, morphometric and sensory results	2000	Questionnaire	no	yes
Nasser Alasseri1. Ahmed Alasraj2. Essam Al-Moraissi3	Minimally invasive genioplasty: an observational study	2022	Face-Q Chin Scale	yes	yes
Wang et al.	Effect of concentrated growth factor on lower lip hypoesthesia after osseous genioplasty: a prospective, split-mouth, double-blind randomized controlled trial	2022	VAS	yes	yes
Wang et al.	Total inferior border ostectomy versus T-shape genioplasty for chin narrowing combined with mandibular contouring	2022	Satisfaction Appearance Score	yes	no
Zhang et al.	Osseous genioplasty: prevention of witch’s chin deformity with no-degloving technique	2021	Face-Q	yes	yes
Hwang, C. H.; Na, Y. S.; Lee, M. C.;	Aesthetic Genioplasty Based on Strategic Categorization	2023	Satisfaction Appearance Score	yes	yes
Tabrizi, R.; Behnia, P.; Kavianipour, M.; Behnia, H.;	Osseous genioplasty versus chin implants: early complications and patient satisfaction	2023	VAS	yes	yes
Xie, Z. Y.; Gao, S.; Yan, K. L.; Lu, T.; Hu, C.; Wang, S.; Shangguan, W. S.; Wu, G. P.;	Correcting the Broad, Flat and Short Chin Using Modified M-genioplasty	2023	Face-Q	yes	yes

## Appendix A

PubMed Session Results (9 August 2023)

**Search**	**Query**	**Items Found**
#5	#3 OR #4	1788
#4	(“orthognat*”[tiab] AND (“Quality of Life”[Mesh] OR “quality of life”[tiab] OR “life qualit*”[tiab] OR “living qualit*”[tiab] OR “quality of living”[tiab] OR “daily life”[tiab] OR qol[tiab] OR hrql[tiab] OR hrqol[tiab] OR ohrqol[tiab]))	305
#3	#1 AND #2	1522
#2	((“Surveys and Questionnaires”[Mesh] OR survey*[tiab] OR questionnair*[tiab] OR “orthognat*”[tiab] OR “telegnat*”[tiab] OR “osteotom*”[tiab] OR “advancement”[tiab]) AND (“Treatment Outcome”[Mesh] OR “Patient Outcome Assessment”[Mesh:NoExp] OR “Patient Reported Outcome Measures”[Mesh:NoExp] OR “Quality of Life”[Mesh] OR “quality of life”[tiab] OR “life qualit*”[tiab] OR “living qualit*”[tiab] OR “quality of living”[tiab] OR “daily life”[tiab] OR qol[tiab] OR hrql[tiab] OR hrqol[tiab] OR ohrqol[tiab] OR “Patient Satisfaction”[Mesh] OR “satisfaction”[tiab] OR “treatment outcome*”[tiab] OR “patient outcome*”[tiab] OR “patient report*”[tiab] OR “patient evaluat*”[tiab] OR “treatment outcome*”[tiab] OR (clinician*[tiab] AND outcome*[tiab]) OR “Visual Analog Scale”[Mesh] OR “Cephalometry”[Mesh] OR “face-Q”[tiab] OR “visual analog scal*”[tiab] OR “visual analogue scal*”[tiab] OR “cephalometr*”[tiab]))	425,006
#1	“Genioplasty”[Mesh] OR “genioplast*”[tiab] OR “mentoplast*”[tiab] OR “chinplast*”[tiab] OR ((“Micrognathism”[Mesh] OR “micrognat*”[tiab] OR “macrognat*”[tiab] OR “microgenia*”[tiab] OR “macrogenia*”[tiab] OR “mandibuloplast*”[tiab] OR “prognat*”[tiab] OR “retrognat*”[tiab] OR “Chin”[Mesh] OR “chin”[tiab] OR “chins”[tiab] OR “mentum”[tiab] OR “mental region”[tiab]) AND (“Orthognathic Surgical Procedures”[Mesh] OR “Orthognathic Surgery”[Mesh] OR “reconstruction*”[tiab] OR “repositioning*”[tiab] OR “reduction*”[tiab] OR “correction*”[tiab] OR “surg*”[tiab] OR “augment*”[tiab]))	7434

Embase.com Session Results (9 August 2023)

**Search**	**Query**	**Items Found**
#6	#5 NOT (‘conference abstract’/it OR ‘conference review’/it)	1728
#5	#3 OR #4	1942
#4	(‘orthognat*’:ab,ti,kw AND (‘quality of life’/exp OR ‘quality of life’:ab,ti,kw OR ‘life qualit*’:ab,ti,kw OR ‘living qualit*’:ab,ti,kw OR ‘quality of living’:ab,ti,kw OR ‘daily life’:ab,ti,kw OR qol:ab,ti,kw OR hrql:ab,ti,kw OR hrqol:ab,ti,kw OR ohrqol:ab,ti,kw))	378
#3	#1 AND #2	1616
#2	((‘questionnaire’/exp OR ‘health survey’/exp OR ‘survey’/exp OR ‘surveys’/exp OR survey*:ab,ti,kw OR questionnair*:ab,ti,kw OR ’orthognat*’:ab,ti,kw OR ‘telegnat*’:ab,ti,kw OR ‘osteotom*’:ab,ti,kw OR ‘advancement’:ab,ti,kw) AND (’treatment outcome’/exp OR ‘quality of life’/exp OR ‘quality of life’:ab,ti,kw OR ‘life qualit*’:ab,ti,kw OR ‘living qualit*’:ab,ti,kw OR ‘quality of living’:ab,ti,kw OR ‘daily life’:ab,ti,kw OR qol:ab,ti,kw OR hrql:ab,ti,kw OR hrqol:ab,ti,kw OR ohrqol:ab,ti,kw OR ‘patient satisfaction’/exp OR ‘satisfaction’:ab,ti,kw OR ‘treatment outcome*’:ab,ti,kw OR ‘patient outcome*’:ab,ti,kw OR ‘patient report*’:ab,ti,kw OR ‘patient evaluat*’:ab,ti,kw OR ‘treatment outcome*’:ab,ti,kw OR (clinician*:ab,ti,kw AND outcome*:ab,ti,kw) OR ‘visual analog scale’/exp OR ‘cephalometry’/exp OR ‘face-Q’:ab,ti,kw OR ‘visual analog scal*’:ab,ti,kw OR ‘visual analogue scal*’:ab,ti,kw OR ‘cephalometr*’:ab,ti,kw))	531,166
#1	’genioplasty’/exp OR ‘genioplast*’:ab,ti,kw OR ‘mentoplast*’:ab,ti,kw OR ‘chinplast*’:ab,ti,kw OR ((’micrognathia’/exp OR ‘micrognat*’:ab,ti,kw OR ‘macrognat*’:ab,ti,kw OR ‘microgenia*’:ab,ti,kw OR ‘macrogenia*’:ab,ti,kw OR ‘mandibuloplast*’:ab,ti,kw OR ‘prognat*’:ab,ti,kw OR ‘retrognat*’:ab,ti,kw OR ‘chin’/exp OR ‘chin’:ab,ti,kw OR ‘chins’:ab,ti,kw OR ‘mentum’:ab,ti,kw OR ‘mental region’:ab,ti,kw) AND (’orthognathic surgery’/exp OR ‘reconstruction*’:ab,ti,kw OR ‘repositioning*’:ab,ti,kw OR ‘reduction*’:ab,ti,kw OR ‘correction*’:ab,ti,kw OR ‘surg*’:ab,ti,kw OR ‘augment*’:ab,ti,kw))	9356

APA PsycInfo (Ebsco) Session Results (9 August 2023)

**Search**	**Query**	**Items Found**
S5	S3 OR S4	4
S4	TI ((“orthognat*” AND (“quality of life” OR “life qualit*” OR “living qualit*” OR “quality of living” OR “daily life” OR “qol” OR “hrql” OR “hrqol” OR “ohrqol”))) OR AB ((“orthognat*” AND (“quality of life” OR “life qualit*” OR “living qualit*” OR “quality of living” OR “daily life” OR “qol” OR “hrql” OR “hrqol” OR “ohrqol”))) OR KW ((“orthognat*” AND (“quality of life” OR “life qualit*” OR “living qualit*” OR “quality of living” OR “daily life” OR “qol” OR “hrql” OR “hrqol” OR “ohrqol”))) OR ((DE “Quality of Life” OR DE “Quality of Life Measures” OR DE “Health Related Quality of Life”) AND (TI (“orthognat*”) OR AB (“orthognat*”) OR KW (“orthognat*”)))	2
S3	S1 AND S2	2
S2	((DE “Surveys” OR DE “Questionnaires”) AND (DE “Treatment Outcomes” OR DE “Patient Reported Outcome Measures” OR DE “Quality of Life” OR DE “Quality of Life Measures” OR DE “Health Related Quality of Life” OR DE “Client Satisfaction”)) OR TI (((“survey*” OR “questionnair*” OR “orthognat*” OR “telegnat*” OR “osteotom*” OR “advancement”) AND (“quality of life” OR “life qualit*” OR “living qualit*” OR “quality of living” OR “daily life” OR “qol” OR “hrql” OR “hrqol” OR “ohrqol” OR “satisfaction” OR “treatment outcome*” OR “patient outcome*” OR “patient report*” OR “patient evaluat*” OR “treatment outcome*” OR (“clinician*” AND “outcome*”) OR “face-Q” OR “visual analog scal*” OR “visual analogue scal*” OR “cephalometr*”))) OR AB (((“survey*” OR “questionnair*” OR “orthognat*” OR “telegnat*” OR “osteotom*” OR “advancement”) AND (“quality of life” OR “life qualit*” OR “living qualit*” OR “quality of living” OR “daily life” OR “qol” OR “hrql” OR “hrqol” OR “ohrqol” OR “satisfaction” OR “treatment outcome*” OR “patient outcome*” OR “patient report*” OR “patient evaluat*” OR “treatment outcome*” OR (“clinician*” AND “outcome*”) OR “face-Q” OR “visual analog scal*” OR “visual analogue scal*” OR “cephalometr*”))) OR KW (((“survey*” OR “questionnair*” OR “orthognat*” OR “telegnat*” OR “osteotom*” OR “advancement”) AND (“quality of life” OR “life qualit*” OR “living qualit*” OR “quality of living” OR “daily life” OR “qol” OR “hrql” OR “hrqol” OR “ohrqol” OR “satisfaction” OR “treatment outcome*” OR “patient outcome*” OR “patient report*” OR “patient evaluat*” OR “treatment outcome*” OR (“clinician*” AND “outcome*”) OR “face-Q” OR “visual analog scal*” OR “visual analogue scal*” OR “cephalometr*”))) OR ((DE “Surveys” OR DE “Questionnaires”) AND (TI (“quality of life” OR “life qualit*” OR “living qualit*” OR “quality of living” OR “daily life” OR “qol” OR “hrql” OR “hrqol” OR “ohrqol” OR “satisfaction” OR “treatment outcome*” OR “patient outcome*” OR “patient report*” OR “patient evaluat*” OR “treatment outcome*” OR (“clinician*” AND “outcome*”) OR “face-Q” OR “visual analog scal*” OR “visual analogue scal*” OR “cephalometr*”) OR AB (“quality of life” OR “life qualit*” OR “living qualit*” OR “quality of living” OR “daily life” OR “qol” OR “hrql” OR “hrqol” OR “ohrqol” OR “satisfaction” OR “treatment outcome*” OR “patient outcome*” OR “patient report*” OR “patient evaluat*” OR “treatment outcome*” OR (“clinician*” AND “outcome*”) OR “face-Q” OR “visual analog scal*” OR “visual analogue scal*” OR “cephalometr*”) OR KW (“quality of life” OR “life qualit*” OR “living qualit*” OR “quality of living” OR “daily life” OR “qol” OR “hrql” OR “hrqol” OR “ohrqol” OR “satisfaction” OR “treatment outcome*” OR “patient outcome*” OR “patient report*” OR “patient evaluat*” OR “treatment outcome*” OR (“clinician*” AND “outcome*”) OR “face-Q” OR “visual analog scal*” OR “visual analogue scal*” OR “cephalometr*”)) OR ((DE “Treatment Outcomes” OR DE “Patient Reported Outcome Measures” OR DE “Quality of Life” OR DE “Quality of Life Measures” OR DE “Health Related Quality of Life” OR DE “Client Satisfaction”) AND (TI (“survey*” OR “questionnair*” OR “orthognat*” OR “telegnat*” OR “osteotom*” OR “advancement”) OR AB (“survey*” OR “questionnair*” OR “orthognat*” OR “telegnat*” OR “osteotom*” OR “advancement”) OR KW (“survey*” OR “questionnair*” OR “orthognat*” OR “telegnat*” OR “osteotom*” OR “advancement”))	84,362
S1	TI (“genioplast*” OR “mentoplast*” OR “chinplast*” OR ((“micrognat*” OR “macrognat*” OR “microgenia*” OR “macrogenia*” OR “mandibuloplast*” OR “prognat*” OR “retrognat*” OR “chin” OR “chins” OR “mentum” OR “mental region”) AND (“reconstruction*” OR “repositioning*” OR “reduction*” OR “correction*” OR “surg*” OR “augment*”))) OR AB (“genioplast*” OR “mentoplast*” OR “chinplast*” OR ((“micrognat*” OR “macrognat*” OR “microgenia*” OR “macrogenia*” OR “mandibuloplast*” OR “prognat*” OR “retrognat*” OR “chin” OR “chins” OR “mentum” OR “mental region”) AND (“reconstruction*” OR “repositioning*” OR “reduction*” OR “correction*” OR “surg*” OR “augment*”))) OR KW (“genioplast*” OR “mentoplast*” OR “chinplast*” OR ((“micrognat*” OR “macrognat*” OR “microgenia*” OR “macrogenia*” OR “mandibuloplast*” OR “prognat*” OR “retrognat*” OR “chin” OR “chins” OR “mentum” OR “mental region”) AND (“reconstruction*” OR “repositioning*” OR “reduction*” OR “correction*” OR “surg*” OR “augment*”)))	85

Web of Science (Core Collection) Session Results (9 August 2023)

**Search**	**Query**	**Items Found**
#5	#3 OR #4	1170
#4	TS=((“orthognat*” AND (“quality of life” OR “life qualit*” OR “living qualit*” OR “quality of living” OR “daily life” OR “qol” OR “hrql” OR “hrqol” OR “ohrqol”)))	450
#3	#1 AND #2	773
#2	TS=(((“survey*” OR “questionnair*” OR “orthognat*” OR “telegnat*” OR “osteotom*” OR “advancement”) AND (“quality of life” OR “life qualit*” OR “living qualit*” OR “quality of living” OR “daily life” OR “qol” OR “hrql” OR “hrqol” OR “ohrqol” OR “satisfaction” OR “treatment outcome*” OR “patient outcome*” OR “patient report*” OR “patient evaluat*” OR “treatment outcome*” OR (“clinician*” AND “outcome*”) OR “face-Q” OR “visual analog scal*” OR “visual analogue scal*” OR “cephalometr*”)))	335,867
#1	TS=(“genioplast*” OR “mentoplast*” OR “chinplast*” OR ((“micrognat*” OR “macrognat*” OR “microgenia*” OR “macrogenia*” OR “mandibuloplast*” OR “prognat*” OR “retrognat*” OR “chin” OR “chins” OR “mentum” OR “mental region”) AND (“reconstruction*” OR “repositioning*” OR “reduction*” OR “correction*” OR “surg*” OR “augment*”)))	6395
